# Foreign

**Published:** 1841-11-06

**Authors:** 


### FOREIGN.

*Fungus Hæmatodes—Amputation.*—The following case, reported by T. Abraham, M. R. C. S. L., possesses considerable interest as proving the rapidity with which fungus hæmatodes occasionally developes itself in distant parts after the almost hopeless attempt to save life by the removal of tumours of this character.

A young lady, aged 20, in the autumn of last year, hurt her knee by a fall, but did not feel much of it at the time, and continued to walk without much inconvenience for about six weeks afterwards, when the joint became very painful on being moved or pressed upon. It gradually enlarged all round, but was not discoloured. Darting and lancinating pains were at length felt in the joint and lower half of the femur, which much harassed and distressed her day and night. In this state I found her on the 15th of January last, when requested to attend the case with Mr. Bateman, who had been previously assisted by Sir Benjamin C. Brodie.

It is not my intention to occupy your space in detailing the treatment; suffice it to say, that the pains were mitigated in a few days but every attempt to cure or suspend the progress of the disease proved useless. The swelling on each side and in front of the joint daily increased, presenting a soft and elastic feel, with an obscure sense of fluctuation. Mr. Bateman and I now considered that, as the disease was progressing, but confined to the limb, and the patient's health rapidly giving way, amputation was the only means of checking it; in this opinion we were afterwards corroborated by Sir B. C. Brodie.

On February the 27th, amputation was performed about two thirds up the femur; this being rendered necessary by the extensive disease of that bone. On laying open the joint after amputation, a large hæmotoid tumour, or substance resembling coagulated blood intersected by cellular strata, was found in it, and the lower half of the femur carious, in which most probably the tumour originated. In about two months the stump was healed, and the patient gradually improved in health, so as to be able for a few weeks to take carriage exercise, and visit her friends. In the beginning of July, however, she was very ill, and it was discovered that effusion into the left side of the chest had taken place. On the 8th instant she expired.

A post mortem examination having been permitted by her friends, has set at rest any doubt that may previously have been entertained as to the nature of the complaint. My friend Mr. Blyth (Mr. Bateman being from home) and I found about two quarts of serum in the left side of the chest, extensive pleuritic adhesions, the *whole* of the left lung converted into an encephaloid mass, weighing about two pounds, of a reddish-white hue, more consistent than brain, and greasy to the touch. The heart (forced into the right side of chest) was small and pale; the parietes thin, the valves sound; the right lung was much compressed, and thickly studded with calcareous deposits. No disease was found in the abdomen.

The above statement is forwarded for publication as additional evidence (if any be wanted) of fungus hæmatodes, or medullary sarcoma, being dependent on a morbid condition of the blood, and of its re-production and rapid growth in another part after it had been removed from its primary seat.—*London Medical Gazette.*

This case reminds us strongly of another occurring in the very commencement of our private practice, which strongly proves the advantage of declining to meddle too hastily with tumours of rapid growth, suspicious character, and doubtful diagnosis, when seen in patients whose constitution is in bad condition, even when the result of a hasty consultation is favourable to an operation, and the ambitious young surgeon is panting for an opportunity to flesh his maiden-knife.

In the winter of 1823–4 we visited, at the request of a very eminent surgeon, a boy of about twelve years of age, whose residence was too distant to permit the gentleman first consulted to assume the charge of the case in the midst of his arduous duties. The boy was pale and feeble, with a scrofulous diathesis, and had been confined to his bed for some weeks, in consequence of apparent inflammation of the hip-joint. From the account given by the mother of the preceding features of the case, and the symptoms present at the time of the first visit, the diagnosis of coxalgia seemed most perfectly plain, and the proper course of treatment for that disease was immediately instituted. On the following day our attention was called to the condition of the left cuspid tooth of the upper jaw, which was said to be aching severely, and the mother wished it extracted. On examination, the tooth was found perfectly sound, but, over the root, there was a small elongated tumour of the gum, extending from its neck nearly as high as the corresponding ala nasi. This tumour very closely resembled an epulis. It was rather broadly and firmly attached to the periosteum, but it was rather unusually elastic, and was the seat of severe lancinating pains, not materially increased by pressure. In the absence of soreness, there was no reason for supposing these pains to originate from inflammation of the periosteum, nor was there any valid reason for attributing them to any ordinary cause of irritation to the nerve. Fearing that the tumour might prove of a malignant and hæmorrhagic character, and knowing the patient to be in a condition which would not bear the exhaustion, we positively prohibited the extraction of the tooth, and ordered opiates combined with antimonials. In the course of a few hours we were sent for in great haste, and found that the tooth had been drawn, and that the patient had already lost many ounces of blood. The boy had fainted repeatedly, and the dentist having exhausted his skill in vain in the endeavour to arrest the bleeding, had fled the house in great alarm. By plugging the cavity with lint steeped in a strong solution of alum, and applying pressure with the finger over the compress for a long time, the hæmorrhage was checked, but not until the life of the child was seriously endangered.

On examining the extracted tooth, the point of the root was found to be surmounted by a portion of soft, ragged, fibrous matter, evidently springing from the periosteum, and torn from a mass of larger dimensions in a line corresponding with the direction of the tooth. Convinced from these circumstances that the morbid structure was connected with the antrum, we earnestly requested a division of responsibility with the high Authority which had been called upon in the first instance.

In the consultation, immediate excision of the tumour was strongly pressed, under the belief that it was periosteal and superficial; and it was with great difficulty, aided by much firmness of opinion, that we accomplished a delay on account of the extreme ill health of the patient, and his obvious inability to endure a formidable operation *if* the tumour should prove to be connected with the antrum.

The case being still treated for coxalgia; in about a week a similar tumour appeared on the left external angular process of the frontal bone, and rapidly grew to the size of a filbert. It was affected with similar lancinating pains. The stomach soon became intolerant of food, vague shooting pains were felt in the abdomen, diarrhœa at length came on, and in about three weeks the patient died in delirium, with slight convulsions.

After death, the antrum was found completely filled with a soft encephaloid mass, the tumour near the external canthus of the eye was found to be much of the same character, though somewhat firmer, a large portion of the right lobe of the liver had undergone an analogous transformation, and the pyloric extremity of the stomach was enormously thickened, and almost reduced to a pultaceous mass! R. C.

---

*On the Application of Oil-varnish to the Immoveable Apparatus, in Fractures of the Limbs of Children.* By M. Tavignot, House-surgeon of the Hospital of Children's Diseases.—When the common immoveable apparatus is applied to the fracture of the lower limb of a young child, at the end of two or three days it becomes perfectly moveable by saturation with urine, and therefore totally incapable of maintaining the fractured parts in apposition. After some trials with glazed silk and spirit-varnish, which were soon permeated with the fluid, M. Tavignot found that oil-varnish answered the purpose extremely well. The best mode of using it is to apply the starch-bandage first, and this having become completely dry, a layer of the varnish is to be smeared over its whole external surface, and also over its superior border and part of its internal surface by a fine brush. The limb is then to be exposed to the air in warm weather, and to artificial heat in winter. Another layer of varnish is to be applied in about two hours, being applied especially on the parts of the limb most exposed to the action of the urine. When the layer has dried, the ordinary coverings of the limb may be replaced, and the urine then glides over the surface of the bandage with great facility without affecting it in the least. M. Tavignot has placed a bandage thus prepared in constant contact with urine, by putting it into a vessel full of this fluid without altering its consistence in any degree.

This is not the only advantage, for the apparatus can be removed without cutting the bandages, by placing the child in a warm bath, for the water insinuates itself between the bandage and the limb, and so softens the former by dissolving some of the starch, that the limb is readily unrolled.

Paper steeped in this varnish appears likely to prove highly useful to the surgeon in protecting parts exposed to the action of acrid or irritating fluids.

[The composition of this varnish is not given. It is probably the common size-varnish of our oil-shops.]—*Brit. and For. Med. Rev.*, *from L'Examinateur Médical.*

---

*Medico-legal Question on the Criminal Production of Abortion in case of an Acephalous Monster.*—On the 2d of June last at the Assizes at Drôme a girl was accused of procuring abortion by criminal means. The fœtus when it came into the world was acephalous; there was no vertebral canal to the spinal column; the spinal marrow and nerves were in a rudimentary state and immediately under the skin. One of the lungs was but half the size of the other, and both sunk in water. The forehead was completely absent, the chin was almost confounded with the chest, and there was scarcely any neck. The fœtus was of about six months. It was proved by MM. Morin and Biguel that it had never respired, and that its life ceased with gestation; but they also remarked on its head a wound and a small ecchymosis which they regarded as a probable result of a criminal manœuvre, executed by the aid of a pointed instrument shortly before abortion, and they thought that the death of the fœtus was the consequence, whilst in the womb of the mother.

The most important question which presented itself, and which was seized by the counsel of the prisoner, was, in a case where the material fact of abortion provoked by the accused was demonstrated, could the penalty apply for delivering herself of a monstrous fœtus, incapable of life, and could a deed be considered a crime which had caused no prejudiee to society nor to an individual? (individualitiè.) The defender after having distinguished abortion from infanticide, which cannot be a crime when the infant is not viable; and alluded to the remarkable fact peculiar to abortion, that the law does not punish the iutention, even when it is manifested by the commencement of execution, argued that it is not the endeavour the law punishes, but the injurious deed alone. "The destruction of a monster incapable of life cannot injure it. The fact, then, cannot constitute the crime of abortion. If it be the deed prejudicial to generation that the law arraigns, it must be said that in this case there existed no prejudice, no crime, and no penalty could be awarded." The jury acquitted the prisoner, notwithstanding the opposition of the *ministre public.*—*Ibid., from Gaz. Med.*

---

*On an unusual variety of Hernia.* By M. Dumeaux.—The following remarkable disposition of parts was observed in the body of a man between 55 and 60 years of age, who had a large scrotal hernia on the right side. After removing all the coverings of the tumour, the hernial sac, or what appeared to be the sac, instead of being a uniformly resisting membrane, presented very distinct muscular fascicula, and the posterior wall of the cæcum was immediately recognized. On examination by the abdomen it was found that the cæcum, instead of occupying the iliac fossa, was placed on the abdominal wall, and covered by peritoneum on one of its surfaces only. Its adherent wall was engaged in the inguinal canal, forming part of the hernial tumour. The free wall, covered with peritoneum followed, being as it were invaginated in the former, and forming towards the abdomen a cavity or true hernial sac, in which seven or eight inches of intestine were engaged.—*Ibid, from Ann. de la Chir.*

